# Nut consumption and risk of atrial fibrillation in the Physicians' Health Study

**DOI:** 10.1186/1475-2891-11-17

**Published:** 2012-03-21

**Authors:** O Khawaja, JM Gaziano, L Djousse

**Affiliations:** 1 Divisions of Aging, Brigham and Women's Hospital and Harvard Medical School, Boston, MA, USA; 2 Massachusetts Veterans Epidemiology and Research Information Center (MAVERIC), Boston Veterans Affairs Healthcare System, Boston, MA, USA; 3 Preventive Medicine, Brigham and Women's Hospital and Harvard Medical School, Boston, MA, USA; 4 Geriatric Research, Education, and Clinical Center (GRECC), Boston Veterans Affairs Healthcare System, Boston, MA, USA

**Keywords:** Nuts, Diet, Atrial fibrillation, Epidemiology, Risk factors

## Abstract

**Background:**

Atrial Fibrillation is highly prevalent in clinical practice affecting approximately 2.3 million people in USA and 4.5 million people in European Union. The aim of the study was to examine the association between nut consumption and incident atrial fibrillation.

**Methods:**

Prospective cohort of 21,054 male participants of Physicians' Health Study I. Nut consumption was estimated using food frequency questionnaire and incident atrial fibrillation was ascertained through yearly follow-up questionnaires. Cox regression was used to estimate relative risks of atrial fibrillation.

**Results:**

The average age was 54.6 ± 9.5 years (40.7-87.1). During a mean follow up of 20 years (median 24 years), 3,317 cases of atrial fibrillation occurred. The crude incidence rate was 7.6, 7.4, 8.2, 7.9, and 6.8 cases/1000 person-years for people reporting nut consumption of rarely/never, 1-3/month, 1/per week, 2-6/week, and ≥ 7/week, respectively. Multivariable adjusted hazard ratios (95% CI) for incident atrial fibrillation were 1.00 (ref), 1.00 (0.90-1.11), 1.09 (0.97-1.21), 1.07 (0.95-1.21), and 0.91 (0.70-1.17) for nut consumption from the lowest to the highest category of nut consumption (p for trend 0.26). No statistically significant association between nut consumption and atrial fibrillation was found when stratified by body mass index (BMI < 25 vs ≥ 25 kg/m^2^) or age (< 65 vs. ≥ 65 years).

**Conclusions:**

Our data did not show an association between nut consumption and incident atrial fibrillation among US male physicians.

## Background

Atrial Fibrillation (AF) is highly prevalent in clinical practice affecting approximately 2.3 million people in USA and 4.5 million people in European Union [1,2]. The incidence of AF increases by about 0.1%-0.2% per year after the age of 40 while AF prevalence ranges from 2%-4% in people over 60 years of age to 8% in people over 80 years of age [3-6]. Prevalence for age- adjusted AF is reported to be higher in men than in women [4,7]. AF is associated with 2-7 fold increased risk of ischemic stroke [8-11] and is also associated with a higher rate of mortality [7,10,11]. Several risk factors of AF have been shown to be influenced by modifiable lifestyle factors. Among dietary factors, nuts are low in sodium and contain many important nutrients including mono- and polyunsaturated fatty acids, fiber, vitamins E, and B_2 _(riboflavin), folate, and essential minerals (magnesium, phosphorus, copper, selenium, and potassium), thereby making them an excellent nutrient source [12]. Nut consumption has previously been associated with improved serum cholesterol [13], blood pressure [14], weight loss [15], risk of diabetes [16] and sudden death/coronary heart disease [17]. Nuts are relatively cheap and widely available and thereby can serve as a cost-effective means to prevent AF. However, it is not known whether nut consumption is associated with the risk of incident AF. Therefore, the current study sought to prospectively assess whether nut consumption is associated with a lower risk of developing AF among US male physicians.

## Methods

### Study population

Data was obtained from the Physicians' Health Study (PHS) I cohort. The details of the PHS I have been described elsewhere [18]. In short, PHS I was a randomized, double blind, placebo controlled trial, of 22,071 US male physicians 40-84 years of age with no history of myocardial infarction, stroke, transient ischemic events or cancer at the time of randomization, with a two-by-two factorial design to study the effects of low dose aspirin (ASA) and beta-carotene on cardiovascular disease and cancer among the US male physicians (1982 to 1995). Of the 22,071 subjects in the PHS, we excluded subjects who were lost to follow up (n = 7) at 12 month post-randomization, those with missing data on nut consumption (n = 612), and those with prevalent AF (n = 398) at 12 months post-randomization. Thus, a final sample of 21,054 participants was selected for current analyses.

Information on health status, risk factors for cardiovascular disease (CVD), dietary, and lifestyle factors was collected by questionnaires. Each participant gave written informed consent and the Institutional Review Board at Brigham and Women's Hospital approved the study protocol.

### Nut consumption

Information on nut consumption was self-reported using a simple abbreviated food frequency questionnaire at 12 months post-randomization (1983-1985). Participants were asked the following: "Please indicate how often, on average, you have eaten each of the following foods during the past year". "Nuts (small packet or 1 oz.)" Possible response categories included "rarely/never", "1-3/month", "1/week", "2-4/week", "5-6/week", "daily", and "2+/day". While the food frequency questionnaire was not validated in the Physicians' Health Study, it has been validated in several cohorts [19-21].

### Primary outcome

Primary outcome was the development of AF in the study population. As presented in the ACC/AHA ESC guidelines for the management of patients with first detected episode of AF it can be classified into: *Paroxysmal *(self terminating usually lasting < 7 days) and *Persistent *(non- self terminating usually lasting > 7 days). *Recurrent *if two or more episodes of AF and permanent AF if Persistent AF is long lasting [1]. All types of AF cases were assessed annually via follow-up questionnaires from 12 months forward. These self- reports of AF have been validated in another study conducted in the same cohort using a more detailed questionnaire on the diagnosis of AF and the review of medical records [22].

### Other variables

Data on demographics, anthropometrics, randomization to ASA, randomization to beta-carotene, diabetes mellitus (DM), hypertension (HTN), hypercholesterolemia, coronary heart disease (CHD), congestive heart failure (CHF) physical activity, smoking as well as cereal, fruit, vegetable, and alcohol consumption were assessed at the baseline (1982-1983). Age was categorized in 5 year categories (< 45, 45-49, 50-54, 55-59, 60-64, 65-69, 70-74, and 75+ y), body mass index (BMI) was classified as lean, overweight, and obese i.e. < 25, 25-29.9, and ≥ 30 kg/m^2^, respectively. Randomization to both ASA and beta-carotene was categorized as yes versus no. For physical activity, participants were asked how often they exercised to sweat; for current analysis, physical activity was dichotomized into exercise to sweat ≥ 1 versus < 1 per week. Smoking was classified as never, past, and current smokers. Alcohol consumption was classified as none, monthly, weekly, and daily. Cereal consumption was classified as ≤ 1, 2-6, and ≥ 7 servings per week. Fruit/vegetable consumption was categorized as servings per week and used as a continuous variable. Diagnosis of DM was made on self-reports via questionnaires mailed to each participant every 6 months during the first year and annually thereafter. HTN was defined as anyone who self reported the diagnosis, BP > 140/90 mm Hg per JNC7 guidelines or on the basis of medication list provided by the study participants. Diagnosis of hypercholesterolemia was also made on self-report as well as on the basis of available blood cholesterol levels. Diagnosis of CHD was made by including patients with self reported history of myocardial infarction (MI) and coronary artery bypass grafting (CABG). CHF diagnosis was again self reported and was verified and validated by detailed review of the medical record [23].

### Statistical analysis

We classified each subject into one of the following categories of nut consumption: rarely/never, 1-3 per month, 1 per week, 2-6 per week, and 7+ per week as described previously in this cohort [36, 37]. We computed person-time of follow up from exposure assessment (12 months post-randomization) until the first occurrence of a) AF, b) death, or c) censoring date i.e. the date of last available follow up. Baseline demographic variables were recorded and compared with respect to each category of nut consumption.

We used Cox proportional hazard models to compute multivariable adjusted hazard ratios (HR) with corresponding 95% confidence intervals (CI) using participants in the lowest category of nut consumption as the reference group. Potential confounding was assessed for established risk factors of AF. The basic model only adjusted for age in 5-year categories. Model 1 was a multivariable model and controlled for age in 5-year categories, BMI randomization to ASA, randomization to beta-carotene, physical activity, smoking, cereal consumption, fruit/vegetable consumption, alcohol consumption, history of HTN, history of hypercholesterolemia, and DM.

In a secondary analysis using BMI of 25 kg/m^2 ^as cut point, we assessed if adiposity modified the relation between nut consumption and AF. We then conducted stratified analyses by BMI < 25 or ≥ 25 kg/m^2 ^and tested statistical interaction using a product term of nut consumption and adiposity variable in a hierarchical model. We also conducted stratified analyses by age using 65 years as cut point to assess if aging modified the relation between nut consumption and AF.

Since information on nut exposure does not describe peanut butter, a relatively large source across the U.S. it was separately queried using the same multivariable model as for nut consumption. We classified each subject into one of the following categories of peanut butter consumption: None, 1-3 per month, 1 per week, 2-6 per week, and 7+ per week. In an additional analysis using the same multivariable model we dichotomized nut consumption into ≥ weekly versus < weekly to see if it modified the relation between nut consumption and AF.

A sensitivity analysis by excluding those who developed any chronic disease such as any cancer, DM, or CVD and consequently might have changed their dietary patterns during the follow-up was also conducted. Assumptions for proportional hazard models were tested (by including main effects and product terms of nut consumption and logarithmic-transformed time factor) and were met (all P values > 0.05). All analyses were conducted using SAS, version 9.2 (SAS Institute, NC). Significance level was set at 0.05.

## Results

Table 1 shows the baseline demographics of 21,054 US male according to the categories of nut consumption. Mean age of the study participants was 54.6 ± 9.5 years (range 40.7 to 87.1 years). Among the total participants reporting nut consumption, there were 20%, 36%, 24%, 17%, and 3% people in the rarely/never, 1-3 per month, 1 per week, 2-6 per week, and ≥ 7 per week categories respectively. Frequent nut consumption was associated with a lower prevalence of current smoking and HTN while a higher prevalence of physical activity, fruit/vegetable intake, and cereal intake. During an average follow up of 20 years (median 24 years), 3,317 new cases of AF were reported. The crude incidence rates of AF were 7.6, 7.4, 8.2, 7.9, and 6.8 cases/1,000 person-years, from the lowest to the highest category of nut consumption (Table 2). There was no statistically significant association between nut consumption and incident AF. Multivariable adjusted hazard ratios (95% CI) for incident AF were 1.00 (ref), 1.00 (0.90-1.11), 1.09 (0.97-1.21), 1.07 (0.95-1.21), and 0.91 (0.70-1.17) for nut consumption of rarely/never, 1-3 per month, 1 per week, 2-6 per week, and ≥ 7 per week with p for linear trend of 0.26 (Table 2).

**Table 1:** Baseline characteristic of 21,054 US male physicians according to nut consumption

Variables	Categories of nut consumption				
	
	Rarely/Never N = 4,273	1-3/month N = 7,654	1/week N = 4,965	2-6/week N = 3,617	≥ 7/week N = 545
**Age (years)**	55.4 ± 9.9	54.3 ± 9.4	54.3 ± 9.3	54.5 ± 9.3	55.9 ± 9.8
**Body mass index (kg/m^2^)**	24.8 ± 2.9	24.8 ± 2.8	24.9 ± 2.8	24.7 ± 2.6	24.1 ± 2.5
**Randomized to ASA (%)**	50.4	49.9	49.2	50.6	50.6
**Randomized to Beta-Carotene (%)**	50.6	49.9	50.2	49.2	50.5
**Hypercholesterolemia (%)**	12.4	11.6	12.2	12.0	12.6
**Hypertension (%)**	26.3	23.9	22.9	21.4	19.0
**Physical activity**	67.2	71.9	74.6	75.5	77.1
**[exercise to sweat ≥ 1 per week] (%)**					
**Current smokers (%)**	12.4	10.9	10.5	10.4	11.8
**Cereal intake [servings per week]**					
**(%)**					
≤ 1	59.5	55.1	51.6	50.8	50.6
2-6	19.9	23.9	26.5	27.0	18.5
≥ 7	20.6	20.9	22.0	22.2	30.8
**Fruit/vegetable (servings/week)**	16.4 ± 9.0	16.4 ± 8.6	16.9 ± 8.4	17.4 ± 8.4	18.8 ± 10.8
**Peanut butter consumption (%)**					
Rarely/Never	65.0	39.8	27.3	27.0	33.0
1-3/month	14.5	31.4	23.9	18.2	22.7
1/week	8.9	13.8	27.5	19.8	14.6
2-6/week	9.3	12.5	19.0	30.8	15.7
≥ 7/week	2.3	2.5	2.4	4.3	14.0
**Current drinking (%)**	83.0	86.0	86.0	85.0	80.7
**Diabetes mellitus (%)**	3.7	2.9	2.9	3.6	5.9
**Congestive heart failure (%)**	0.2	0.1	0.1	0.1	0.0
**Coronary heart disease (%)**	1.5	1.0	1.0	0.9	0.6

Data are presented as mean ± standard deviation or percentages

**Table 2:** Hazard ratios (95% CI) for atrial fibrillation according to nut consumption in Physicians' Health Study

Nut intake	Cases/person- years	Crude incidence rate (1,000 person-years)	Hazards Ratio (95% Confidence Interval)		
			Unadjusted	Age adjusted*	Model 1**
**Rarely/Never**	644/84926	7.6	1.0	1.0	1.0
**1-3/month**	1170/157704	7.4	0.97 (0.88 - 1.06)	1.01 (0.92 - 1.12)	1.00 (0.90 - 1.11)
**1/week**	842/102290	8.2	1.07 (0.97 - 1.19)	1.12 (1.01 - 1.24)	1.09 (0.97 - 1.21)
**2-6/week**	587/74642	7.9	1.03 (0.92 - 1.15)	1.05 (0.94 - 1.18)	1.07 (0.95 - 1.21)
**≥ 7/week**	74/10865	6.8	0.90 (0.71 - 1.14)	0.88 (0.69 - 1.12)	0.91 (0.70 - 1.17)
**P for trend**			0.41	0.33	0.26

*Age as ordinal variable Adjusted for age (< 45, 45-49, 50-54, 55-59, 60-64, 65-69, 70-74, 75+ y)

**Adjusted for Age (< 45, 45-49, 50-54, 55-59, 60-64, 65-69, 70-74, 75+ y), body mass index (< 25, 25-29.9, and 30+ kg/m^2^), aspirin, beta-carotene, physical activity (1 or more times per week vs. < 1 per week), smoking (never, past and current smokers), cereal servings per week (≤ 1, 2-6, ≥ 7), fruit/vegetable servings per week (continuous), alcohol consumption (none, monthly, weekly, daily), history of hypertension, history of hypercholesterolemia and history of diabetes

In a secondary analysis, nut consumption was not associated with incident AF among lean subjects (BMI < 25 kg/m^2^) [multivariable adjusted hazard ratios (95% CI) of 1.0 (reference), 1.07 (0.92-1.23), 1.16 (0.99-1.36), 1.16 (0.98-1.37), and 0.97 (0.70-1.34) from the lowest to the highest category of nut consumption, respectively (p for trend 0.11)]. Corresponding values for individuals that were overweight/obese were 1.0 (reference), 0.95 (0.82-1.09), 1.02 (0.87-1.19), 0.98 (0.82-1.18), and 0.84 (0.54-1.29), respectively (p for trend 0.95)].

Similarly, nut consumption was not associated with incident AF among younger (age < 65 years) physicians [multivariable adjusted hazard ratios (95% CI) of 1.0 (reference), 0.99 (0.88-1.11), 1.06 (0.94-1.20), 1.07 (0.94-1.23), and 0.92 (0.69-1.22) across consecutive categories of nut consumption, respectively (p for trend 0.30)]. Corresponding values for older participants (≥ 65 y) were 1.0 (reference), 1.06 (0.84-1.34), 1.20 (0.93-1.53), 1.04 (0.78-1.37), and 0.88 (0.48-1.60), respectively (p for trend 0.71)].

In a separate analysis multivariable adjusted hazard ratios (95% CI) for incident AF for peanut butter consumption were 1.00 (ref), 1.01 (0.91-1.11), 1.13 (1.02-1.25), 1.01 (0.90-1.12), and 1.00 (0.80-1.25) from the lowest to the highest category, respectively (overall p = 0.41). The multivariable adjusted hazard ratios (95% CI) for incident AF using nut consumption as ≥ weekly versus < weekly was 1.07 (0.99-1.15).

Results of sensitivity analysis excluding those who developed any chronic disease such as any cancer, DM, or CVD did not alter the results either [hazard ratios (95% CI) of 1.0 (reference), 1.00 (0.89-1.14), 1.07 (0.94-1.23), 1.10 (0.95-1.27), and 0.94 (0.70-1.26) from the lowest to the highest category of nut consumption, respectively, p for trend 0.22].

## Discussion

Our findings do not support a meaningful association between the consumption of nuts and incident AF among apparently healthy US male physicians. In addition, adiposity or advancing age did not modify the relation between nut intake and AF risk. To the best of our knowledge, this is the first large prospective study to assess the association between nut consumption and the incidence of AF.

Several factors could have lead to this null association between nut consumption and incident AF. We did not have any data on the type of nuts consumed. We were also unable to characterize different types of nut preparations like salted, roasted, etc to study their effects on the incident AF. Perhaps in 1980s, nut intake was not necessarily low in sodium and this could have lead to a higher prevalence of HTN, an important risk factor for AF. However, lower prevalence of HTN with increased nut consumption argues against it as a possible explanation. Each of the different types of nut (i.e. walnut, pistachio, hazelnut, etc) has been demonstrated to have a specific nutrient profile. Another important limitation was a single assessment of nut consumption during the study period. The results indicate that although the power was limited at the levels of nut consumption that would be most likely to influence AF risk, there was a non-statistically significant increase in AF risk among all consumers of nuts weekly or more. As a post hoc analysis, this would therefore be an interesting analysis for other data sets. We did not have information on family history of hypertension, hypercholesterolemia, or diabetes and therefore were unable to adjust for it. The crude way of assessing physical activity was also a potential limitation in our analysis and could have lead to residual confounding. Lastly our population consisted of only male physicians, who might have a higher consumption of nuts due to being aware of their health benefits making it difficult to generalize our findings to the general population.

Despite a lack of association between nut consumption and AF, beneficial effects of nuts have been reported on numerous outcomes considered to be important risk factors for developing AF. Nuts have been shown to improve serum cholesterol levels [13], lower blood pressure [14,24-28], lower the risk of DM [16], improve weight loss [15], improve inflammation [29], and lower the risk of cardiovascular mortality [17]. Our study has several strengths, including a large sample size, 20 years follow up, use of a standardized questionnaires, and a homogeneous group of male physicians able to recognize signs and symptoms of AF than the general population.

## Conclusion

In summary, our study does not provide evidence in support of a significant association between nut consumption and incident AF among US male physicians.

## Competing interests

The authors declare that they have no competing interests.

## Authors' contributions

Each author has participated sufficiently, intellectually or practically, in the work to take public responsibility for the content of the article, including the conception, design, and conduct of the experiment and for data interpretation. OK participated in study design, carried out collection of data, data analyses and drafted the manuscript. JG helped in drafting the manuscript along with providing significant advice and consultation. LD conceived of the study, participated in study design, helped in drafting the manuscript and provided significant advice and consultation. All authors read and approved the final manuscript.
